# A Trimeric Lipoprotein Assists in Trimeric Autotransporter Biogenesis in Enterobacteria*[Fn FN2]

**DOI:** 10.1074/jbc.M113.513275

**Published:** 2013-12-25

**Authors:** Iwan Grin, Marcus D. Hartmann, Guido Sauer, Birte Hernandez Alvarez, Monika Schütz, Samuel Wagner, Johannes Madlung, Boris Macek, Alfonso Felipe-Lopez, Michael Hensel, Andrei Lupas, Dirk Linke

**Affiliations:** From the ‡ Max Planck Institut für Entwicklungsbiologie, 72076 Tübingen,; the § Institut für Medizinische Mikrobiologie and Hygiene and; the ¶ Proteome Center Tübingen, Universität Tübingen, 72076 Tübingen, and; the ‖ Abteilung Mikrobiologie, Fachbereich Biologie/Chemie, Universität Osnabrück, 49076 Osnabrück, Germany

**Keywords:** Bacterial Adhesion, Bacterial Pathogenesis, Crystal Structure, Lipoprotein, Membrane Trafficking, Enterobacteria, Gram-negative Bacteria, Membrane Insertion, Autotransport

## Abstract

Trimeric autotransporter adhesins (TAAs) are important virulence factors of many Gram-negative bacterial pathogens. TAAs form fibrous, adhesive structures on the bacterial cell surface. Their N-terminal extracellular domains are exported through a C-terminal membrane pore; the insertion of the pore domain into the bacterial outer membrane follows the rules of β-barrel transmembrane protein biogenesis and is dependent on the essential Bam complex. We have recently described the full fiber structure of SadA, a TAA of unknown function in *Salmonella* and other enterobacteria. In this work, we describe the structure and function of SadB, a small inner membrane lipoprotein. The *sadB* gene is located in an operon with *sadA*; orthologous operons are only found in enterobacteria, whereas other TAAs are not typically associated with lipoproteins. Strikingly, SadB is also a trimer, and its co-expression with SadA has a direct influence on SadA structural integrity. This is the first report of a specific export factor of a TAA, suggesting that at least in some cases TAA autotransport is assisted by additional periplasmic proteins.

## Teaser

**Background:** Autotransporter adhesins reach the bacterial cell surface by a complex mechanism.

**Results:** In the case of the autotransporter SadA from *Salmonella*, a lipoprotein assists in surface display.

**Conclusion:** The similarity to eukaryotic MATH domains suggests that the lipoprotein assists in trimerization of SadA.

**Significance:** Understanding the similarities between autotransport systems might lead to new ways of inhibiting bacterial adhesion.

## Introduction

The outer membrane of Gram-negative bacteria forms the outermost barrier between the bacterial cell and the outside world. As such, the role of the outer membrane is astoundingly complex. It acts as a protective barrier against harmful substances such as antibiotics, bacteriocins, and, especially for pathogenic bacteria, also against factors of the host immune system. At the same time it permits interaction with the outside, such as uptake of nutrients, export of secreted factors, as well as sensing and adhesion. These very different functions are typically performed by transmembrane β-barrel proteins, the major protein family present in the outer membrane. This diverse group of evolutionarily related integral membrane proteins (1, 2) is characterized by its three-dimensional structure, a cylinder formed by antiparallel β-strands spanning the outer membrane and connected by loops on either side. The pore inside of a β-barrel allows the passage of small molecules such as ions or nutrients but also protein domains or whole proteins (3).

A special class of β-barrel proteins are monomeric and trimeric autotransporter proteins, commonly referred to as type Va and type Vc secretion systems, respectively (4, 5). These are large proteins (often of 3000 residues or more) consisting of a translocator domain, a typically 12-stranded barrel that is inserted into the outer membrane, and a passenger part, which is exported to the bacterial surface through the pore formed by the translocator domain. The translocator domain acts as an export pore as well as an anchor, tethering the exported passenger to the bacterial surface; in several cases of monomeric autotransporters, the passenger domain is proteolytically cleaved and released into the extracellular space post-translocationally (6). In the case of trimeric autotransporters, each monomer of the homotrimer contributes four strands to the barrel, through which then all three passengers are exported.

Although the passengers of monomeric autotransporters can be structurally and functionally rather diverse, trimeric autotransporter passengers are trimeric coiled-coil structures interspersed with a limited number of domains, which are thought to modulate the flexibility of the otherwise rigid fiber, or provide adhesion to abiotic surfaces, biopolymers (*e.g.* collagen or fibronectin), and host cell surface structures. The ratio of coiled-coil segments to other domains varies dramatically between different trimeric autotransporters. *Yersinia* YadA consists of a single extended coiled coil with only one head domain at its end, and others such as *Haemophilus* Hia have a low content in coiled-coil segments (7). With adhesion being their main function, this group of proteins is commonly referred to as trimeric autotransporter adhesins (TAAs)^6^ (8).

The biogenesis of monomeric and trimeric autotransporter adhesins is in large part similar to that of other outer membrane proteins. Both groups have an N-terminal signal peptide targeting them for export into the periplasm by the Sec machinery. A first challenge arises from the fact that the β-barrel domain is typically at the very C terminus of the polypeptide, the part that is translocated last. During this time, the passenger must be kept from (mis-)folding or aggregating in the periplasm. This role is performed by periplasmic chaperones such as Skp and SurA as well as the chaperone/protease DegP. Additionally, in the case of TAAs, which are translocated into the periplasm as monomers, trimerization of the β-barrel segments must occur. The Bam complex then catalyzes the insertion of the β-barrel domain into the outer membrane, upon or during which the passenger is exported, yielding the mature protein (4).

During previous work on the *Salmonella* trimeric autotransporter adhesin SadA, we noted that in the enterobacterial genera *Escherichia*, *Salmonella,* and *Shigella,* the chromosomal location of *sadA* is conserved between the *mtl* operon for mannitol metabolism and the *lld* operon for l-lactate metabolism (9). Further investigation revealed that the adhesin forms an operon with a small predicted lipoprotein (STM3690 in *Salmonella enterica*) encoded directly upstream of the adhesin gene. We named this lipoprotein SadB in *S. enterica*. In this study, we determined the three-dimensional structure of SadB using x-ray crystallography and show that SadB enhances the surface display of SadA, suggesting a direct involvement of SadB in the autotransport mechanism of the trimeric autotransporter adhesin SadA.

## MATERIALS AND METHODS

### 

#### 

##### Strains, Plasmids, Primers, and Sequence Data

Sequence information relevant for this work was retrieved from NCBI as follows: SadB, AAL22549.1/gi|16422256; YajI, YP_488704.1/gi|388476518. Strains (Table 1), primers (Table 2), and plasmids (Table 3) used in this study are listed below.

**TABLE 1:** **Strains used in this study**

Designation	Relevant characteristics	Source or Ref.
*E. coli* TOP 10	Cloning strain	Invitrogen
*S. enterica* serovar *typhimurium* NCTC12023	Wild type	NCTC
MvP681	ΔSTM3691::aph	This study
MvP682	ΔSTM3691::FRT	This study
MvP788	aph araC PBAD::STM3690	This study
MvP789	FRT araC PBAD::STM3690	This study
MvP790	aph araC PBAD::STM3691	This study
MvP791	FRT araC PBAD::STM3691	This study

**TABLE 2:** **Primers used in this study**

Designation	Sequence 5′ to 3′
Fwdlipo	ATACACGGTCTCAAATGCACAAAAATGGAAAATTTATCCC
Revlipostop	ATACACGGTCTCAGCGCTTTATTTTTTTGGCTTCTTTTTTGTATCG
RevSadAStop	ATACACGGTCTCAGCGCTTTACCACTGGAAGCCCGC
FwdlipoSol	ATGGTAGGTCTCAAATGAGTGATTACTTCGCAGATAAACACC
STM3691-Del-For	ATAATAGCCATTGATACAATTATTTTAGAAAAGGAAATTAGTGTAGGCTGGAGCTGCTTC
STM3691-Del-Rev	TGCCATTGCCTTTGATGTCGGGAGTGTTGTTACTTCATTACATATGAATATCCTCCTTAG
STM3960-Red-BAD-For	TATTTTTATAAAGCATTGCTATGAGCAATTGATAAATAACGTGTAGGCTGGAGCTGCTTCG
STM3690-Red-BAD-Rev	CCAACGCAAGCAGGGGGATAAATTTTCCATTTTTGTGCATGGTTAATTCCTCCTGTTAGC
STM3691-Red-BAD-For	TAACGATACAAAAAAGAAGCCAAAAAAATAATAGCCATTGTGTAGGCTGGAGCTGCTTCG
STM3691-Red-BAD-Rev	CCGTAGCGGCATTCCAGAGGACTTTAAATATTCTATTCATGGTTAATTCCTCCTGTTAGC
STM3690-RedBAD-Check	GGTTGTGTTGATAATCGTAGC
STM-Ctrl-For	AAAGGTCACCGAAGTCGTTG
STM-Ctrl-Rev	GGCAACATAGCCTTTCAGC

**TABLE 3:** **Plasmids used in this study**

pASK IBA3	Expression vector	IBA, Germany
pASK IBA3 SadB	*sadB* in pASK-IBA3	This study
pASK IBA3 SadB ΔSignal peptide	*sadB*Δ*1–22* in pASK-IBA3	This study
pASK IBA2-SadA	*sadA* in pASK-IBA2	40
pASK IBA3 SadBA	*sadBA* in pASK-IBA3	This study
pKD46	Expression of Red recombinase	19
pCP20	Expression of FLP recombinase	19
pBAD-Myc-HisB	Expression vector *araC* P*_BAD_*	Invitrogen
p2795	Basis vector for *aph* cassette	21
p3253	*araC* P*_BAD_* in p2795	This study
pWRG435	pFPV.25.1 mTagRFP	Roman G. Gerlach

##### Bioinformatics

The genomic context of *sadB* and its paralogs was investigated using GCView (10) in the MPI Bioinformatics Toolkit (11). Sequences homologous to SadB or YajI were collected from up to three rounds of PSI-BLAST (12) and forwarded to GCView for genomic context lookup and inspection. Genes upstream and downstream of the gene of interest were selected for further iterations of GCView to verify the conservation of the genomic context.

##### DALI

Upon determination of the three-dimensional structure of SadB, the model was submitted to the Dali server (13) to search for structurally similar proteins. The query consisted of either the full model or just the C-terminal domain (residues 90–213).

##### Cloning

All primers for pASK IBA vectors were designed using Primer D'Signer 1.1 software.

The *sadB* gene from *S. typhimurium* was cloned into pASK-IBA3 using primers Fwdlipo and Revlipostop. For cytosolic expression, a construct of *sadB* without the N-terminal signal peptide was created using primers FwdlipoSol and Revlipostop. The *sadBA* operon was cloned into pASK-IBA3 using primers Fwdlipo and RevSadAStop.

##### Protein Expression and Purification

Cultures were grown at 37 °C in LB medium supplemented with 0.1 mg/ml ampicillin to an *A*_600_ = 0.6–0.8. Protein expression was subsequently induced by adding 0.2 μg/ml anhydrotetracycline (AHTC). After 4 h, cells were harvested by centrifugation. Cell pellets were resuspended in 20 mm Mops/NaOH, pH 6.5, 50 mm NaCl, 10 mm MgCl_2_ containing protease inhibitor mix (Roche Applied Science) and a pinch of DNase I (Applichem). Bacteria were lysed using a French press. Cell debris and membranes were pelleted by ultracentrifugation at 200,000 × *g* for 45 min at 8 °C. The supernatant was diluted with Buffer A (20 mm Mops/NaOH, pH 6.5, 1 mm EDTA) and loaded onto a cation exchange column (Source S, GE Healthcare). In the case of the SadB construct containing the native lipid anchor, 1% *n*-octyl-polyoxyethylene (Bachem, Buchs, Switzerland) was added to all chromatography buffers. Bound protein was eluted using a linear salt gradient of 0–1 m NaCl in Buffer A. Fractions containing SadB were identified using SDS-PAGE and pooled. The pooled fractions were purified to homogeneity on a Sephadex S75 size exclusion column (GE Healthcare) equilibrated in 20 mm Mops/NaOH, pH 6.5, 50 mm NaCl. Purified protein was stored at 4 °C.

##### Antibody Purification

Rabbit anti-SadA antibody was described before (9). Rabbit anti-SadB antibody was raised using purified SadB in an in-house facility. The obtained polyclonal serum was affinity purified on a 1-ml HiTrap NHS-activated HP Column (GE Healthcare) according to the manufacturer's manual. Purified SadB was coupled to the column to be used as bait. Antibodies were eluted with 1 m NaCl, 1 m MgCl_2_, and 4 m MgCl_2_. Only the 4 m MgCl_2_ fraction was used for all subsequent experiments.

##### Cell Shaving

Cells were grown in LB medium supplemented with 0.1 mg/ml ampicillin at 37 °C to an *A*_600_ = 0.6. Protein expression was induced by addition of 0.2 μg/ml AHTC for 2 h. 1 × 10^9^ cells were harvested by centrifugation in a tabletop centrifuge, washed once with PBS, and then resuspended in 100 μl of PBS. 0.2 units of proteinase K were added, and samples were incubated for 10, 30, or 60 min at 37 °C. After incubation, the reaction was stopped by addition of protease inhibitor mix (Roche) and vigorous mixing. Whole cells were spun down in a tabletop centrifuge. The supernatant was analyzed by mass spectrometry as described below.

##### SDS-PAGE and In-gel Digestion

The supernatants from cell shaving with proteinase K were submitted to a gel run on a one-dimensional SDS-PAGE (NuPAGE 12% precast BisTris gels, Invitrogen). Each gel lane was cut in eight equally sized slices (vertical axis) for in-gel digestion. The proteins were subjected to tryptic in-gel digestion as described previously (14). The resulting peptide mixtures were desalted with C18 Stage Tips before LC/MS measurement.

##### Liquid Chromatography-Mass Spectrometry (MS) Analysis

Mass spectrometry analysis of the complete, lipidated SadB lipoprotein was performed using an ion trap (HCTultra PTM Discovery, Bruker Daltonics) equipped with a nano-ESI source (Proxeon Biosystems). LC-MS analysis of in-gel digests was performed on a nano-LC (Easy-nLC, Thermo Fisher Scientific) coupled to an LTQ-Orbitrap-XL (Thermo Fisher Scientific) through a nano-LC-MS interface (Proxeon Biosystems), as described previously (14). Peptides were eluted using a segmented gradient of 5–90% HPLC solvent B (80% acetonitrile in 0.5% acetic acid) at a flow rate of 200 nl/min over 57 min. MS data acquisition was conducted in the positive ion mode. The mass spectrometer was operated in the data-dependent mode to automatically switch between MS and MS/MS acquisition. Survey full-scan MS spectra were acquired in the mass range of *m*/*z* 300–2000 in the Orbitrap mass analyzer at a resolution of 60,000. An accumulation target value of 10^6^ charges was set, and the lock mass option was used for internal calibration (15). The 10 most intense ions were sequentially isolated and fragmented in the linear ion trap using collision-induced dissociation at the ion accumulation target value of 5000 and default collision-induced dissociation settings. The ions already selected for MS/MS were dynamically excluded for 90 s. The resulting peptide fragment ions were recorded in the linear ion trap.

##### Data Processing and Analysis

Raw files were processed using the MaxQuant software (version 1.2.2.9) (16). Raw MS spectra were first processed by the Quant module to generate peak lists. To retrieve peptide sequences from the processed spectra, the integrated Andromeda peptide search engine (17) was utilized. The processed MS spectra were searched against a decoy *Salmonella enterica* subsp. *typhimurium* LT2 database (Uniprot organism 99287 reference proteome as of December 2, 2013) containing 4536 forward protein entries plus the sequences of 248 commonly observed contaminants.

In the database search, carbamidomethylation (Cys) was set as fixed modification, whereas oxidation (Met) and acetylation (protein N termini) were set as variable modifications. The mass tolerances for precursor and fragment ions were set to 6 ppm and 0.5 Da, respectively. A false discovery rate of 1% was set at the peptide, protein level.

##### Subcellular Localization

To determine the subcellular localization of SadB density gradient centrifugation, method 5 described by Thein *et al.* (18) was used. Briefly, cells expressing SadB with its native signal peptide were grown overnight at 30 °C without addition of AHTC, harvested, and then lysed as described above. The leakiness of the expression system was sufficient to generate usable amounts of natively localized SadB. After ultracentrifugation, the supernatant was discarded, and the membrane pellet was resuspended in 1 ml of 10 mm Tris/HCl, pH 7.0, 15% (w/w) sucrose, 5 mm EDTA. A 30–55% (w/w) continuous sucrose gradient was prepared on a Biocomp Gradient Station (Fredericton, New Brunswick, Canada) in 13-ml centrifuge tubes (SW 41 Ti, Beckman Instruments). All sucrose solutions contained 10 mm Tris/HCl, pH 7.0, 5 mm EDTA. The sample was carefully layered on top of the gradient and centrifuged at 250,000 × *g* for 12–16 h. After centrifugation, the gradient was split into 1-ml fractions that were analyzed by SDS-PAGE and subsequent Western blotting with αSadB antibody. αOmpX (2) and αYidC were used as markers for outer and inner membrane fractions, respectively. α-Rabbit DyLight 800-conjugated antibodies (Pierce) were used as secondary antibody. The membranes were scanned on an Odyssey infrared imaging system and analyzed using Image Studio 2 (LI-COR Biosciences, Lincoln, NE).

##### Flow Cytometry

Cells were grown in LB medium supplemented with 0.1 mg/ml ampicillin at 37 °C to an *A*_600_ = 0.6. Protein expression was induced by addition of 0.2 μg/ml AHTC for 2 h. 1 × 10^9^ cells were harvested by centrifugation in a tabletop centrifuge, washed with 1% BSA in PBS, and stained with affinity-purified rabbit αSadA (see above) in 1% BSA/PBS for 1 h at 4 °C and subsequently with allophycocyanin-conjugated secondary antibody (1:200, Jackson ImmunoResearch) in 1% BSA/PBS for 1 h at 4 °C in the dark. Surface localization of SadA was measured by flow cytometry in a BD Biosciences LSR II. Measurements were analyzed using WinMDI (J. Trotter) software. Data are means for three independent experiments.

##### Fluorescence Microscopy

Samples for immunofluorescence microscopy were prepared using the protocol for FACS (above). Cy3-conjugated goat IgG anti-rabbit IgG (Jackson ImmunoResearch) was used as secondary antibody. After immunolabeling, cells were immobilized on poly-l-lysine-coated coverslips, stained with 0.4 μg/ml DAPI for 10 min in the dark, embedded in Mowiol-DABCO, and examined under a Zeiss Axioplan microscope with an EXFO X-Cite 120 excitation light source.

##### Generation of S. enterica Strains

*S. enterica* serovar *typhimurium* strain NCTC 12023 was used as wild-type strain, and other *Salmonella* strains used in this study are isogenic (Table 1). A deletion strain in *sadA* (STM3691) was generated by λ Red recombinase-mediated recombination basically as described before (19). For the generation of strains with expression of *sadBA* or only *sadA* under control of the P*_BAD_* promoter of the arabinose operon, we used Red recombineering (20, 21). pBAD-myc HisB was digested with NdeI and SacI, and a fragment containing *araC* and P*_BAD_* was recovered and subcloned in p2795 (21). The resulting plasmid p3253 served as template vector for generation of a promoter cassette consisting of *aph* flanked by FRT sites, *araC* and P*_BAD_*. p3253 was amplified by PCR using STM3960-Red-BAD-For/Rev or STM3961-Red-BAD-For/Rev for chromosomal integration of the P*_BAD_* promoter cassette upstream of *sadBA* or *sadA*, respectively.

The proper insertion of the promoter cassette was controlled by PCR using check primers listed in Table 2. If required, the *aph* resistance gene was removed by FLP-mediated recombination. The functionality of the P*_BAD_* promoter cassette was confirmed by chromosomal integration upstream of *phoN* and determination of phosphatase activity in response to induction by arabinose (data not shown).

##### Analysis of SadA Surface Expression in Salmonella

*Salmonella* strains harboring the P*_BAD_* promoter cassette upstream of *sadA* or *sadBA* were grown in LB medium overnight and subcultured by 1:31 dilution in LB supplemented with 0.4% glucose or 0.4% arabinose for repression or induction of P*_BAD_*, respectively. Cultures were grown at 37 °C with aeration for 4 h. The absorbance of bacterial cultures was adjusted to *A*_600_ of 0.2 in PBS, and 50 μl of this suspension were dropped on coverslips with 0.02% poly-l-lysine and allowed to dry at room temperature. Subsequently, bacteria were fixed with 3% paraformaldehyde in PBS for 60 min at 37 °C. Fixed samples were washed three times with PBS, and washing was repeated after each incubation step. Samples were stained with a 1:100 dilution of rabbit-α-SadA in blocking solution (PBS, goat serum 2%, BSA 2%) and incubated for 1 h at RT. Bound antibody was labeled with α-goat-α-rabbit Alexa488. Bacteria harbored pWRG435 for constitutive expression of mTagRFP. Finally, samples were mounted on glass slides with Fluoroprep (BioMerieux) and sealed with Entellan (Merck). Stained samples were kept at 4 °C until observation.

##### Quantification of SadA Surface Expression

Stained samples of *Salmonella* were observed with a Cell Observer® Zeiss microscope with an ×100 objective and N.A. of 1.54. Excitation of Alexa488 was performed by LED illumination at 10% intensity; DAPI was exited with a mercury lamp and the respective excitation/emission filter. Z stacks were acquired from various fields of view with an Axiocam® Zeiss. Images were then processed and analyzed with ZEN 2012. For quantification of the signal of SadA, regions of interest were scored for at least 100 individual bacteria.

##### Phage Display

Phage display experiments were performed using the Ph.D.-7 and Ph.D.-12 Phage Display Libraries from New England Biolabs following the manufacturer's manual. Purified SadB (cytosolic construct without signal peptide) was used as bait. After three rounds of panning, phage DNA from up to 50 clones was isolated (QIAprep Spin M13 kit, Qiagen) and sequenced.

##### X-ray Crystallography

For crystallization, the protein was concentrated to 8 mg/ml in 20 mm MOPS/HCl, pH 7, 50 mm NaCl. Crystallization trials were performed at 20 °C in 96-well sitting-drop vapor diffusion plates with 50 μl of reservoir solution and drops containing 400 nl of protein solution in addition to 400 nl of reservoir solution. The best diffracting crystals were obtained within 1 week with a reservoir solution containing 0.2 m lithium sulfate, 0.1 m Tris, pH 8.5, and 20%(w/v) PEG 4000. Single crystals were transferred into a cryoprotectant drop containing reservoir solution supplemented with 10% (v/v) PEG 400 before flash-cooling in liquid nitrogen. For experimental phasing, crystals were soaked overnight in a drop containing reservoir solution supplemented with 5 mm K_2_PtCl_4_ prior to cryoprotection and flash-cooling. A native and a derivative dataset were collected at beamline X10SA (PXII) at the SLS (Paul Scherrer Institute, Villigen, Switzerland) at 100 K using a PILATUS 6M detector (DECTRIS). Diffraction images were processed and scaled using the XDS program suite (22). Using SHELXD (23), four strong platinum sites were identified. After density modification with SHELXE, the resulting electron density map could be traced by Buccaneer (24) to a large extent. The model was completed by cyclic manual modeling with Coot (25) and refinement with Phenix (26). Analysis with Procheck (27) showed excellent geometries for the final structure. Data collection and refinement statistics are summarized in Table 4.

**TABLE 4:** **Data collection and refinement statistics** Values in parentheses refer to the highest resolution shell. The Ramachandran plot statistics show the percentage of residues in the most favored/additionally allowed/generously allowed/disallowed regions, respectively, as defined and determined using the program Procheck (27).

	Native	K_2_PtCl_4_ derivative
Wavelength	1.0 Å	1.071 Å
Space group	H3	H3
Cell dimensions	*a* = *b* = 118.45, *c* = 159.18	*a* = *b* = 118.53, *c* = 158.84
Resolution	40 to 2.45 Å (2.60 to 2.45 Å)	40 to 3.0 Å (3.18 to 3.0 Å)
Completeness	99.7% (99.2%)	99.8% (98.7%)
Redundancy	4.0 (3.9)	20.9 (19.9)
*I*/σ(*I*)	19.20 (1.86)	34.95 (3.92)
*R*_merge_	4.2 (67.9)	7.2 (87.3)
*R*_cryst_/R_free_	20.8/25.0%	
Ramachandran plot statistics	93.8/5.8/0.2/0.2%	

For visual comparison against the structures of homologous proteins (PDB codes 1CZY and 2JWY) the secondary structure matching algorithm (28) as implemented in Coot (25) was used. For 1CZY, the three β domains were individually superimposed on the three β domains of SadB. In 2JWY, the first model of the NMR ensemble was superimposed on each of the three β domains of SadB.

## RESULTS AND DISCUSSION

### 

#### 

##### Bioinformatics

**FIGURE 1.**
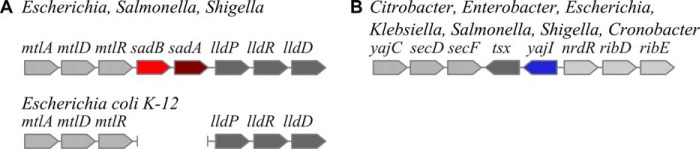
**Genomic context of *sadB* and *yajI* is conserved in Enterobacteria.** *A, sadBA* operon is located between the *mtl* operon and the *lld* operon. *E. coli* K12 has a 5-kb deletion encompassing the *sadBA* operon. *B, sadB* paralog *yajI* is located between the *yajC-secDF* operon and the putative *nrdR-ribDE-nusB* operon. Note that although *sadB* and *sadA* are linked in an operon, *yaiJ* and *tsx* are not.

Detailed analysis of the neighboring genes of *Salmonella* trimeric autotransporter adhesin *sadA* revealed an open reading frame encoding a small predicted periplasmic lipoprotein upstream of *sadA*, which is conserved in *Salmonella, Shigella,* and *Escherichia*. Markedly, in enterobacterial strains in which the adhesin was lost, such as the laboratory strain *E. coli* K12, the deletion also encompasses the lipoprotein gene, such that the *mtl* operon is directly followed by the *lld* operon, with both operons remaining intact (Fig. 1*A*). This, together with the short intergenic distance of 44 bp in *Salmonella,* leads us to hypothesize that the gene coding for the lipoprotein, which we subsequently call *sadB* and *sadA,* forms an operon.

Sensitive sequence homology searches using HHPred (29) against the PDB revealed only one known structure of a similar protein, a paralog that exists in Enterobacteria (YajI in *E. coli*, 14% sequence identity, 41% sequence similarity). The gene *yajI* is also found in a conserved genomic location, between the *yajC-secDF* operon and the putative *nrdR-ribDE-nusB* operon. Many species also have the *tsx* gene in the same location; this codes for an outer membrane β-barrel nucleotide transporter (which is not an autotransporter, Fig. 1*B*). The two genes do not appear to form an operon based on their intergenic distance of ∼300 bp. The available NMR structure of YajI (2JWY) only covers the C-terminal domain of the lipoprotein. No functional data are available for either of the paralogous proteins.

##### SadB Is an Inner Membrane Lipoprotein

**FIGURE 2.**
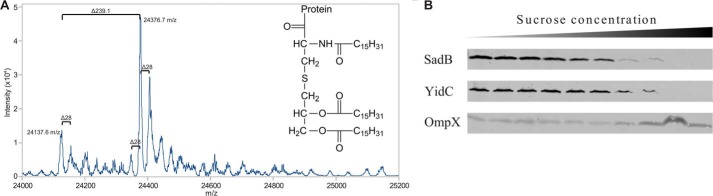
**SadB is a periplasmic inner membrane lipoprotein.** *A,* mass spectrometry analysis of full-length SadB with native lipid anchor. The main mass peak at 24376.7 Da corresponds to the expected molecular weight of the protein with an tri-palmityl anchor (see *inset*). The lighter mass at 24,137.6 Da is the result of the loss of one of the palmityl chains by hydrolysis. Mass differences of 28 Da (-C2H4-) result from the incorporation of stearic acid (C18:0) or myristic acid (C14:0) instead of palmitic acid (C16:0) in the lipid anchor. *B,* subcellular localization of SadB by density gradient centrifugation. In a sucrose gradient SadB is found in the lower density band corresponding to the inner membrane. As control, OmpX (an outer membrane β-barrel protein) is only found in the higher density band corresponding to the outer membrane.

SadB was predicted to be an inner membrane lipoprotein by LipoP and ClubSub-P (30, 31) due to an aspartic acid in the +2 position of the predicted cleavage site. To verify the prediction, we overexpressed *sadB* from a plasmid. Very mild induction was used to avoid overloading cellular secretion and signal peptide processing mechanisms and to ensure native localization of SadB; in our hands, massive overexpression led to partial inclusion body formation, improper processing of the signal peptide, and mislocalization. For the experiments shown in Fig. 2, the very low leaky expression of the vector was used (no extra inducing agent was added to the culture medium). Membrane fractionation (18) showed that SadB is found in the lower density membrane band of the sucrose gradient, corresponding to localization in the inner membrane (Fig. 2*B*). Mass spectrometry of SadB isolated from membrane preparations further confirmed the cleavage of the signal peptide and attachment of a canonical tripalmityl (C16:0) anchor to the N-terminal cysteine residue of the protein, with minor mass peaks originating from stearic acid (C18:0, mass increase by 28 Da) or from myristic acid (C14:0, mass decrease by 28 Da) replacing individual palmityl residues (Fig. 2*A*). We can conclude from these results that SadB is, as predicted, a lipoprotein of the inner membrane, protruding into the periplasmic space of the bacteria.

##### SadB Enhances the Surface Display of SadA in E. coli

As noted above, SadB has no homologs of known function. The genomic association with *sadA* suggests a functional linkage, which is supported by the localization in the periplasm, where SadA is transiently localized on its way to the cell surface. We therefore hypothesized that SadB is involved in the biogenesis of SadA by an unknown mechanism. To investigate whether SadB has any effect on the export of SadA to the surface, we created inducible overexpression constructs for the whole operon or for *sadA* alone. As the native expression conditions of the *sadBA* operon are unknown, we did not use the original promoter.

**FIGURE 3.**
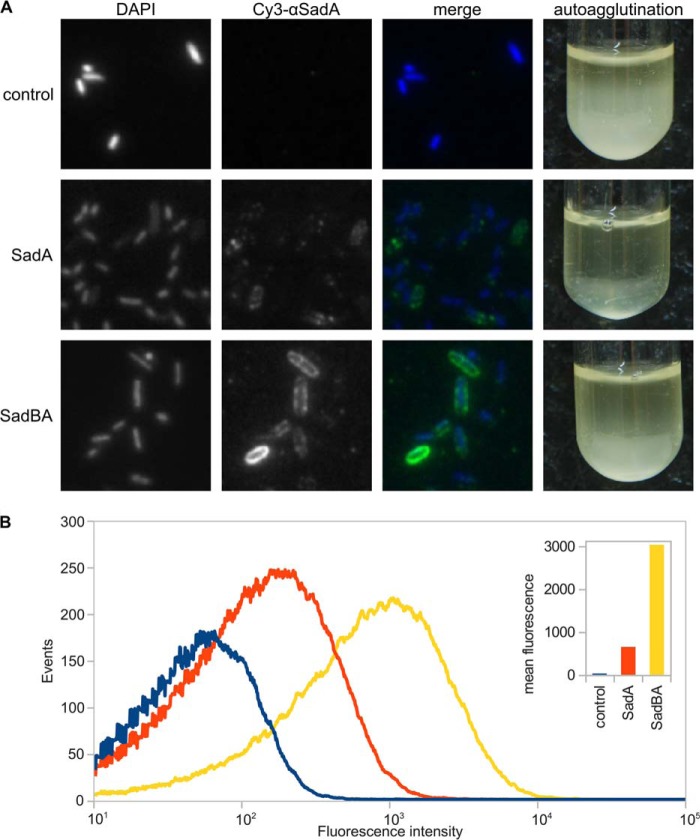
**SadB enhances the surface display of SadA and reduces autoagglutination.** *A,* immunofluorescence microscopy and cell aggregation assay of bacteria expressing *sadA*, *sadBA,* or an empty vector control. Surface-localized SadA was stained using a specific antibody. *B,* flow cytometry analysis of bacteria expressing *sadA*, *sadBAm*, or an empty vector control. Surface-localized SadA was stained using a specific antibody. The *inset* shows the mean fluorescence intensity for each sample.

After staining of whole bacteria with αSadA antibody, immunofluorescence microscopy showed a strong signal over the whole surface of bacteria expressing *sadBA*. Bacteria lacking SadB displayed a much weaker fluorescence signal (Fig. 3). Flow cytometry using cells stained with an αSadA antibody confirmed that bacteria expressing *sadBA* have a 4–6-fold higher mean fluorescence over cells expressing only *sadA*, suggesting a significantly higher amount of SadA on the cell surface (Fig. 3*B*). In line with our hypothesis, this observation can be explained by accumulation of SadA in the periplasm in the absence of the SadB or degradation of incompletely translocated SadA by periplasmic proteases such as DegP.

When expressing *sadA* alone, we noticed that the cells formed denser, stickier pellets after centrifugation, which were hard to resuspend. This was confirmed in an autoagglutination assay, where cells were left to settle down after induction of protein expression. After 8–12 h the supernatant of cultures expressing *sadA* was clear, with all bacteria on the bottom of the tube, whereas cultures expressing *sadBA* and controls were still turbid (Fig. 3). Cell viability was unaffected. A possible explanation for this effect is misfolding of SadA on the cell surface, which leads to exposed hydrophobic surfaces in the protein, by which cells then aggregate. This would suggest that SadB supports the biogenesis of well formed SadA trimers.

**FIGURE 4.**
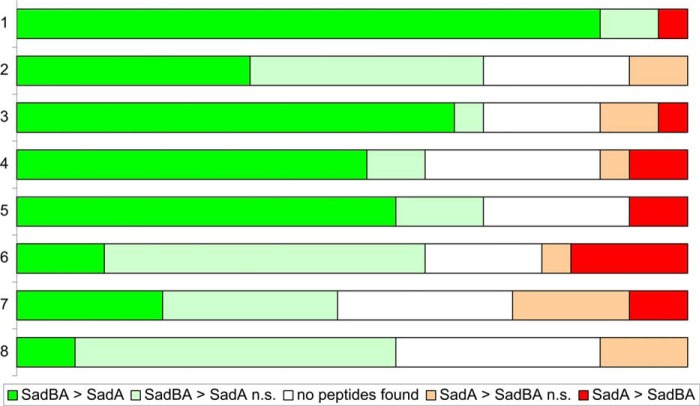
**Mass spectrometry of SadA after cell shaving with proteinase K (partial digest).** Most peptides of SadA are more abundant in samples where the whole operon was expressed, compared with samples where only *sadA* was expressed, in line with previous findings that SadB enhances SadA surface display. Furthermore, SadA is more susceptible to proteolysis in the absence of SadB based on the observation that the largest fragments are only found in SadBA samples. Supernatants from cell shaving with proteinase K were separated on SDS-PAGE. Each gel lane was cut into eight equally sized slices (vertical axis, where *1* represents the slice with the highest molecular weight of fragments, and *8* represents the lowest), and the peptides in each slice were analyzed by mass spectrometry. In total, 23 peptides specific to SadA were identified. For each slice, peptide intensities were compared between samples with SadBA or SadA after normalizing for total protein amount. Peptides were grouped depending on which sample the intensity of the peptide was higher. A 4-fold difference was considered significant (*dark red* and *green bars*). Peptides with a lower difference (below the arbitrary significance cutoff level) were considered not significant (*n.s.*) and are shown as *light red* and *green bars*, for reference. Sometimes the individual peptides were not detected in a particular lane (*white bar*).

To analyze if SadB leads to improved folding and thus protease stability of SadA, we performed a cell shaving assay using proteinase K and analyzed the resulting fragments by mass spectrometry (Fig. 4 and details for detected peptides and mapping onto the SadA sequence can be found in supplemental S1 and S2). After 10 min of incubation with the protease, we found a significantly higher fraction of peptides from high molecular weight fragments in the SadBA sample compared with the sample where only SadA was expressed after normalization for total protein amount; in other words, SadA was partially digested in both samples but was more easily and quickly broken down to smaller fragments in the absence of SadB. This finding supports the notion that SadB indeed improves the protease resistance of SadA and suggests a direct effect of SadB on the folded state of the SadA fiber. Based on the knowledge that SadA contains highly repetitive sequence motifs (9), especially six consecutive repeats of 70–120 amino acids in the stalk of SadA, which have between 55 and >90% identity, it is tempting to assume that SadB could help to define the register of the three exporting SadA chains that form the final trimer by synchronizing the export.

##### SadB Is Required for Proper Surface Expression of SadA in S. enterica

**FIGURE 5.**
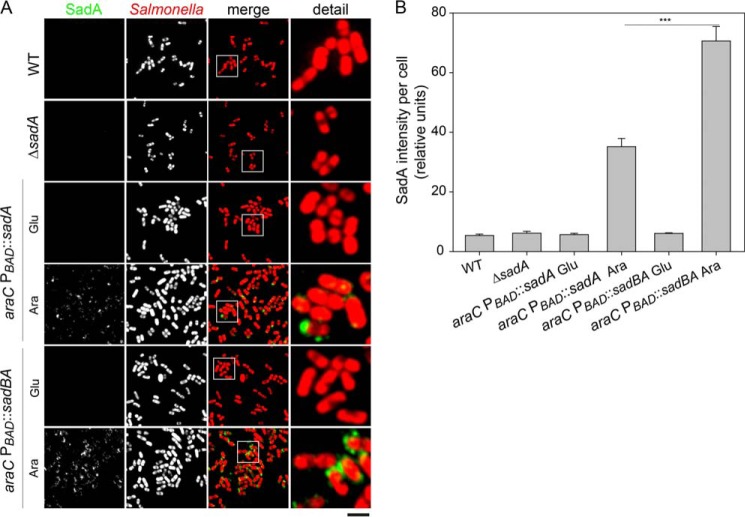
**Role of SadB in surface expression of SadA by *S. enterica*.** *A,* inducible expression of *sadBA* or *sadA* in *Salmonella*. WT, Δ*sadA,* and recombinant strains bearing the inducible arabinose promoter P*_BAD_*::*sadBA* or P*_BAD_*::*sadA* were grown for 4 h in LB with 0.4% glucose or arabinose as indicated, immobilized on coverslips with 0.02% poly-l-lysine, immunostained, and observed by confocal laser scanning microscopy. SadA was detected by immunostaining (*green*). Bacterial cells were labeled mTagRFP. *Scale bar,* 5 μm (overview) and 1 μm (detail). *B,* surface expression of SadA is reduced in absence of SadB. The immunofluorescence signals of SadA from each strain shown in *A* were quantified using ZEN 2012. At least 100 bacteria were scored. Statistical analysis was performed with the Student's *t* test (***, *p* < 0.001).

To show that the observed SadB effect is relevant also in a more native setting, we investigated the role of SadB in *S. enterica*. Various growth conditions were used, but none of these conditions resulted in expression of SadA detectable by Western blotting or immunofluorescence of bacterial cells. This observation is in line with the previous studies indicating that most *Salmonella* adhesins are not expressed under culture conditions (32). To obtain an experimental system that allows analysis of SadB function in *Salmonella*, we generated strains with *sadBA* or only *sadA* under control of the inducible promoter P*_BAD_*. Upon induction with arabinose, synthesis of SadA was observed for both P*_BAD_*::*sadBA* and P*_BAD_*::*sadA* strains. However, the analysis of surface expression showed that signals for SadA were reduced if only *sadA* was expressed in *Salmonella* (Fig. 5). Although the surface localization of SadA was diffuse for the P*_BAD_*::*sadA* strain, the P*_BAD_*::*sadBA* strain showed that a clustered distribution of SadA with several foci per cell were found with high signal levels. Neither *sadA* nor *sadBA* expression in *Salmonella* resulted in macroscopic autoaggregation. However, microscopic inspection indicated that *sadBA* expression, but not expression of *sadA* alone, resulted in formation of small clusters of SadA-positive cells (Fig. 5). This again suggests a direct involvement of SadB in the biogenesis of functionally active SadA.

We anticipate that the localization of SadA in clusters might be required for the proposed function as an adhesin. However, the exact role of SadA in *Salmonella* adhesion and pathogenesis is still unknown, although it has been reported to promote weak adhesion to eukaryotic cells and biofilm formation (33).

##### Direct Interaction of SadB with SadA Cannot Be Shown

**FIGURE 6.**
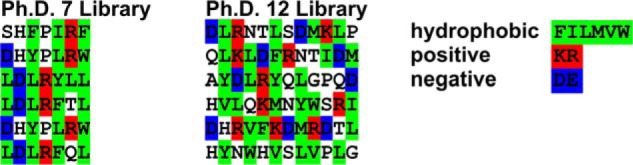
**Representative peptides selected by phage display against SadB.**

To assay whether SadB could recognize and specifically bind unfolded peptides with similarity to the SadA sequence, we used a phage display assay with SadB as bait with a library of random 7-mer or 12-mer peptides. The phage display experiment did not converge on a specific sequence. Rather, the recovered sequence motifs in general showed an alternating pattern of hydrophilic and hydrophobic residues (for examples see Fig. 6), somewhat similar but not strikingly identical to sequence motifs from the head, coiled-coil stalk, and membrane anchor of SadA and other trimeric autotransporter proteins. Also, chemical cross-linking experiments coupled to antibody pulldown assays failed to show an interaction of SadB with SadA *in vivo* (data not shown), suggesting that the interaction is weak and transient.

##### Structure of SadB

**FIGURE 7.**
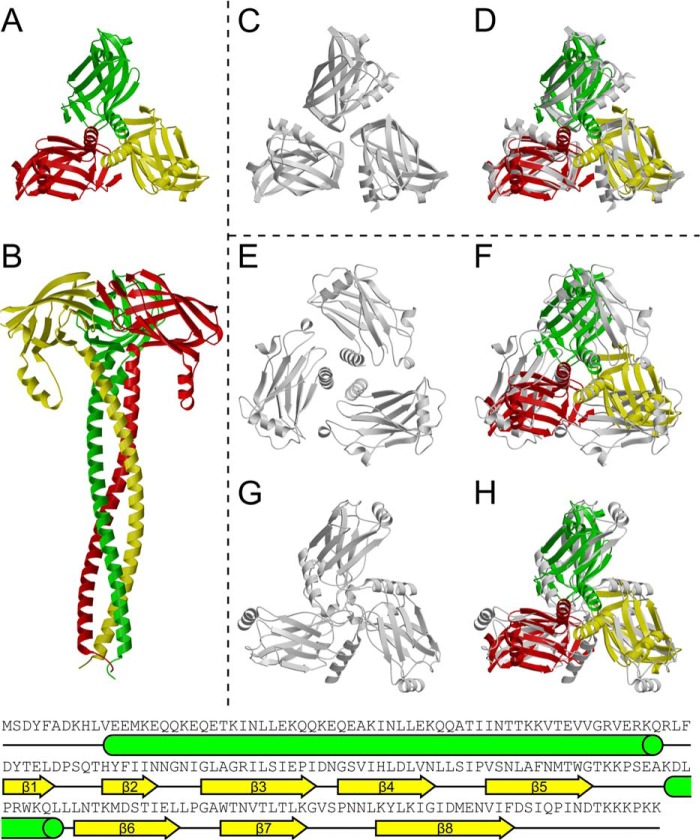
**Structure of SadB compared with the structures of TRAF2 and YajI.** *A* and *B,* structure of SadB viewed alongside and perpendicular to the axis of the coiled coil, colored by monomer. *C* and *D,* superposition of SadB (*colored*) with the YajI structure from PDB entry 2JWY (*gray*). The YajI C domain is superimposed on the three C domains of SadB (r.m.s.d. 1.9, 2.0, and 2.0 Å for 101, 102, and 102 aligned CA positions, respectively). *E–H,* superposition of SadB (*colored*) with the TRAF2 structure from PDB entry 1CZY (*gray*). *C* and *D,* superposition is based on the coiled coil, and in *E* and *F,* individual monomers of the Traf2 C domains are superimposed on the individual C domains of SadB (r.m.s.d. 3.2, 3.2, and 3.0 Å for 99, 105, and 99 aligned CA positions, respectively). It is apparent that the orientation of the C domains with respect to the coiled coil is different in the two proteins.

To obtain the structure of SadB, a construct lacking the N-terminal signal peptide and replacing the lipid-modifiable cysteine residue Cys-22 by a serine residue was created. The resulting protein was solubly expressed in the cytosol without the lipid anchor. Crystallization trials yielded crystals diffracting to 2.45 Å. After initial attempts to solve the structure via molecular replacement with the NMR structure of YajI (2JWY) were not fruitful, we were able to solve it via single isomorphous replacement using a platinum derivative. Like SadA, SadB is a homotrimer (Fig. 7, *A* and *B*); it is held together by an extended N-terminal coiled coil of nine heptads, which leads into three separate globular C-terminal domains of β-sandwich topology, each composed of two antiparallel β-sheets. In this variant of the Ig fold, the first sheet has the strand order β-1, β-8, β-5, and β-6, whereas the loop connecting β-5 and β-6 is especially long and includes a short α-helix protruding toward the N terminus. The second sheet has strand order β-2, β-3, β-4, and β-7. In tracing the electron density of this domain, the YajI structure was very helpful as it has the identical topology (Fig. 7, *C* and *D*).

Trimerization of the protein was not observed during protein purification, including size exclusion chromatography, suggesting a low trimerization propensity of the coiled coil. Notably, the paralogous protein YajI was described as a monomer in the PDB database (2JWY), although sequence analysis suggests the presence of an N-terminal coiled coil, probably because it does not readily trimerize in solution either. We ascribe this to the high number of polar residues in core positions of the coiled-coil domain. Of the 18 core residues of each SadB protomer, six are glutamine, four in position *a* of the heptad repeat and two in position *d*. Of these, four are arranged within two closely spaced segments with 10 consecutive polar residues each, which provide very little local hydrophobicity for coiled-coil assembly. Indeed, in the N-terminal half of the coiled coil, 22 of 28 residues are polar and 14 are charged. Polar core residues are known to lower the stability and thus the oligomerization propensity of coiled coils (34) and to play, for example, a prominent role in the coiled-coil segments of SadA (35). Trimerization of SadB is therefore expected to be dependent on elevated local concentrations of the protein as would happen *in vivo* on the outside of the inner membrane, to which the protein is tethered by its lipid anchor.

##### SadB Is Topologically Similar to the Eukaryotic MATH Domain

**FIGURE 8.**
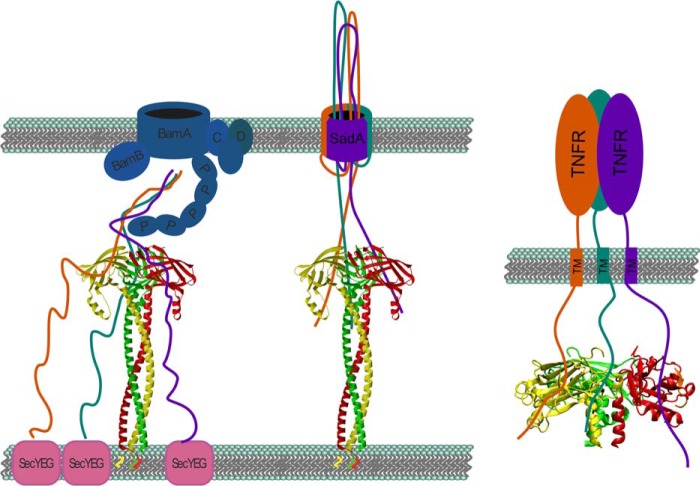
*Left*, proposed mode of action for SadB. SadB could be involved in initial stages (*left side*) and/or later stages (*right side*) of SadA membrane insertion and autotransport. *Right,* TRAF2 binding to TNF receptor (adapted from McWhirter *et al.* (36)). The trimeric MATH domain interacts with the three unfolded peptide chains of the TNF receptor. We propose an analogous mechanism where SadB supports the trimerization of unfolded SadA.

A DALI search using the C-terminal domain of SadB (residues 90–213) revealed that, beside the already known similarity and clear homology to YajI (with a Z-score of 10.6 and an r.m.s.d. of 2.9 Å over 116 aligned residues), SadB shows high structural similarity to a number of bacterial proteins that are also variants of the Ig fold. All of these proteins display a different β-strand topology as shown by the shorter alignable region (typically ∼80–90 residues), and in most cases they do not have an N-terminal coiled coil and are typically of unknown function. Interestingly, however, the structure is also highly similar to the C-terminal domain of human TNF receptor associated factor 2 (TRAF2) (PDB codes 1CZY and 1QSC and similar to Fig. 7, *E–H*), which is called the Meprin and TRAF homology (MATH) domain. This similarity between SadB and MATH domains is undetectable on the sequence level, yet the topology of β-strands in the C domain is identical between SadB, YaiI, and all known MATH domain structures. The DALI Z-scores range from 5.8 to 6.2, and the r.m.s.d. from 3.1 to 3.2 Å, over 101–103 residues. MATH domains are known to bind peptides from the cytoplasmic domain of TNF receptors across an interface on the outer β-sheet. The receptors trimerize upon binding to trimeric TNF, which positions the cytoplasmic domains in a triangle fit for binding to the MATH domains of a TRAF2 trimer. This mode of binding prefers trimeric, ligand-bound receptor complexes over single receptor molecules by means of increased avidity of three binding sites over one (36, 37). Even though an evolutionary link between SadB and the MATH domains of Traf proteins cannot be established based on sequence analysis, the striking structural similarity and the fact that both act as a trimer on trimeric membrane-bound proteins suggest a comparable mode of action to bring together or bind three unstructured protein chains of a second trimeric protein (Fig. 8).

##### Conclusion

SadB is a trimeric lipoprotein located in the inner membrane of *Salmonella* spp., with homologs in other enterobacteria, including pathogenic *E. coli* species. The *sadAB* operon is conserved in Enterobacteriaceae. A second paralogous protein of unknown function (YajI) exists in almost all enterobacteria but is not linked to an operon or autotransporter.

The reduced amount and increased stickiness of surface-localized SadA in the absence of SadB and the decreased resistance of SadA to proteinase K in the absence of SadB all suggest a direct influence of SadB on SadA export and folding. If indeed trimeric SadB can bind three nascent SadA polypeptide chains in the periplasm after or during Sec-dependent secretion, it might directly influence the (trimeric) autotransport process. We assume that such an interaction would be weak and transient, also from the fact that cross-linking, pulldown, and phage display experiments performed in this study were not conclusive. A weak interaction close to the inner membrane, and thus to the Sec machinery where the SadA chain is extruded to the periplasm, would presumably help to synchronize the export of three SadA chains, avoiding out-of-register interactions of the highly repetitive, long, and at that stage unfolded polypeptides, as displayed in Fig. 8. A longer retention of unfolded passenger domains at the bacterial inner membrane to achieve productive autotransport has also been suggested for the unusually long signal peptides frequently found in autotransporters (38, 39). SadB could take an analogous role in the case of SadA. It seems that SadB is a specific invention of enterobacteria to help in the autotransport of a specific class of TAAs, and whether other analogous “helper” systems exist for other autotransporter systems will be an interesting subject for future research.

## Footnotes

6 The abbreviations used are:

TAA: trimeric autotransporter adhesin
BisTris: 2-[bis(2-hydroxyethyl)amino]-2-(hydroxymethyl)propane-1,3-diol
PDB: Protein Data Bank
AHTC: anhydrotetracycline.
